# *EGFR* Amplification and Glioblastoma Stem-Like Cells

**DOI:** 10.1155/2015/427518

**Published:** 2015-06-02

**Authors:** Katrin Liffers, Katrin Lamszus, Alexander Schulte

**Affiliations:** ^1^Laboratory for Brain Tumor Biology, Department of Neurosurgery, University Medical Center Hamburg-Eppendorf, 20246 Hamburg, Germany; ^2^Laboratory for Tumor Genetics, Department of Neurology, University Medical Center Hamburg-Eppendorf, 20246 Hamburg, Germany

## Abstract

Glioblastoma (GBM), the most common malignant brain tumor in adults, contains a subpopulation of cells with a stem-like phenotype (GS-cells). GS-cells can be maintained *in vitro* using serum-free medium supplemented with epidermal growth factor, basic fibroblast growth factor-2, and heparin. However, this method does not conserve amplification of the Epidermal Growth Factor Receptor (*EGFR*) gene, which is present in over 50% of all newly diagnosed GBM cases. GS-cells with retained *EGFR* amplification could overcome the limitations of current *in vitro* model systems and contribute significantly to preclinical research on EGFR-targeted therapy. This review recapitulates recent methodological approaches to expand stem-like cells from GBM with different *EGFR* status in order to maintain EGFR-dependent intratumoral heterogeneity *in vitro*. Further, it will summarize the current knowledge about the impact of *EGFR* amplification and overexpression on the stem-like phenotype of GBM-derived GS-cells and different approaches to target the EGFR-dependent GS-cell compartment of GBM.

## 1. Introduction

Glioblastoma (GBM) is the most common malignant brain tumor in adults. Despite advances in surgical procedures and therapeutic options, the live expectancy of GBM patients has remained poor with a median survival of only 12–15 months [1]. Although GBMs are characterized by extensive intra- and intertumoral heterogeneity at the histological and molecular level, they can be divided into four major subtypes based on their global expression profiles associated with distinct prognosis [2, 3]. According to Verhaak et al., these are the mesenchymal, neural, proneural, and classical subtypes, each defined by specific genetic aberrations or expression of marker genes (mesenchymal: NF1; neural: SYT1; proneuronal: PDGFR*α*/IDH1, classical: EGFR).

Pediatric and adult GBMs contain a subpopulation of cells with a stem-like phenotype (GBM stem-like cells; GS-cells), identified by the cell surface marker CD133 (also termed Prominin-1) [4–8]. Similar to adult neural stem cells (NSCs), GS-cells contain the ability to self-renew and to differentiate along neural lineages, that is, astrocytes, neurons, and oligodendrocytes, when cultured in differentiation medium (fetal calf serum, retinoic acid, and cyclic adenosine monophosphate) [5, 9]. For GS-cells, the ability to initiate tumors that recapitulate the heterogeneous phenotype of their parent tumor when implanted into the brain of immunodeficient mice is considered the central criterion to distinguish GS-cells from nonstem-like tumor cells.

At the molecular level, GS-cells in neurosphere cell culture have been described to closely mirror the genotype and the transcriptional phenotype of primary GBM tissue as opposed to conventional adherent monolayers which were established in the presence of serum [10–15]. This is also reflected in the conservation of the molecular subtypes of GBM in GS-cells, while these are lost in conventional cell lines [2, 14, 16, 17].

GS-cells were found to be highly resistant to radio- and chemotherapy* in vitro* and* in vivo* [4, 8, 18] and to adapt rapidly to changes in the tumor microenvironment, that is, acidic stress [19] or hypoxia [20, 21]. Data from our lab could further demonstrate that GS-cells undergo a metabolic switch from glycolysis to the pentose phosphate pathway in response to hypoxia, resulting in decreased proliferation and increased migration [22]. This indicates an inherent metabolic plasticity, translated into phenotypic properties such as migration or proliferation, in order to adapt to microenvironmental oxygen changes. Finally, these mechanisms might also contribute to treatment resistance.

Clinically, a stem-cell related gene expression signature in patient-derived tumors (self-renewal signature [23]) was found to be associated with resistance to radio/chemotherapy in GBM patients [24]. Additionally, a high proportion of cells positive for putative GS-cell markers such as CD133, nestin, or PDPN was a negative prognostic factor for progression-free survival (PFS) and overall survival (OS) in GBM patients [11, 25–28]. This has led to an investigation of GS-cell targeted therapies (reviewed in [29–31]), including differentiation therapies [9, 32], oncolytic therapies with CD133-targeted measles virus [33], or indirect targeting of the perivascular GS-cell niche [20, 34, 35].

The most frequent genetic alteration in GBMs is an amplification of the Epidermal Growth Factor Receptor (*EGFR*) gene and/or its overexpression at the protein level, which is present in 40–60% of all GBM cases [36, 37]. Half of the amplified cases additionally express a constitutively active, oncogenic EGFR deletion variant lacking the ligand-binding domain (exons 2–7) termed EGFRvIII [38, 39]. EGFR/EGFRvIII expression is associated with increased proliferation and migration of GBMs, contributing to the malignant phenotype of these tumors in an angiogenesis-independent manner [40–42]. Additionally, expression of EGFRvIII has been found to promote and accelerate angiogenesis in preclinical GBM models* in vivo* [43, 44]. However, therapeutic targeting of the EGFR by inhibiting tyrosine kinase activity or by interfering with ligand-induced activation has not improved overall life expectancy for GBM patients when compared to standard treatment [45–48].

One of the major drawbacks for the analysis of the impact of* EGFR* amplification on targeted therapy is that it is rapidly lost when cells from* EGFR*-amplified GBM are taken into culture [49]. As a result of this limitation, preclinical models for studying EGFR biology in GBM largely relied on ectopic overexpression of EGFR and/or EGFRvIII in nonamplified GBM cell lines and a subsequent blockade of the overexpressed proteins [50–52]. Over the years, different methods have been developed to overcome these limitations and to maintain* EGFR* amplification in addition to a stem-like phenotype* in vitro*, which allowed for the investigation of the contribution of the EGF/EGFR axis to a glioma stem cell phenotype in an* EGFR*-amplified background.

## 2. Glioblastoma Cells with a Stem-Like Phenotype* In Vitro*

Different approaches have been described to isolate and to expand GS-cells from GBM tissue* in vitro* based on phenotypic criteria or marker expression. Using cell culture conditions originally developed to promote* in vitro* growth of neural precursor cells from the neurogenic subependymal zone (serum-free medium supplemented with epidermal growth factor (EGF) and basic fibroblast growth factor (bFGF)), Ignatova et al. described cells with stem-like features isolated from cortical glial tumors (anaplastic astrocytoma, WHO grade III and GBM, and WHO grade IV) [7]. Phenotypically, cells selected under these conditions grew as neurospheres with a heterogeneous cellular morphology, were clonogenic, and expressed neural lineage markers such as nestin and glial fibrillary acidic protein (GFAP).

Using a similar approach, Galli et al. isolated stem-like cells from glioblastoma tissue which, in addition to their phenotypic analogy to neural precursor cells, established tumors upon orthotopic xenotransplantation in nude mice [4].

Pollard et al. described glioma stem cells propagated as adherent cultures on a laminin matrix using growth factor-supplemented neurosphere medium in the absence of serum, thereby preventing differentiation [53]. These cells exhibited stem-like features* in vitro* and also initiated tumors that recapitulated the cellular heterogeneity of primary GBM.

An alternative approach to isolate tumor stem-like cells is based on biological properties of these cells and enriches the “side population” of dissociated tumor tissue or established tumor cell lines, including glioblastoma [54, 55]. Here, GS-cells are identified by their high efflux capacity for chemical dyes like Hoechst 33342 due to the high expression of drug resistance-related ABC-transporters like ABCG2 [56–58]. The side population of GBM cell lines has been shown to contain cells with stem-like properties [54, 59, 60]. However, this approach is presently challenged since a side population could not be detected in neurospheres derived from primary GBM tissue [61]. Furthermore, Golebiewska et al. could demonstrate that the side population derived directly from primary GBM tissue mostly contains brain endothelial cells and is nontumorigenic* in vivo* upon xenotransplantation [62].

Singh et al. isolated stem-like cells from GBM by enrichment of CD133-positive cells from primary tumor material. They could show that as little as 100 CD133-positive cells initiated a tumor upon orthotopic injection, while 100.000 CD133-negative cells did not, delivering key evidence that CD133^+^ cells are glioma stem-like cells. Based on these findings, CD133 has since been the most widely used marker for identifying GS-cells and is so far the most reliable molecule for isolation and/or identification of GS-cells. However, the idea of a restrictive model where CD133 expression defines GS-cells is currently under debate [63]. For example, expression of CD133 is subject to changes in the tumor microenvironment such as hypoxia, indicating that CD133 might be a marker for bioenergetic stress [14, 22, 64]. Additionally, different reports suggest that CD133-negative cells can also exhibit stem-like characteristics, most importantly the capacities for self-renewal and tumor initiation* in vivo* [53, 65]. A comprehensive overview of the complex regulation of CD133 in GBM is provided by Campos and Herold-Mende in [66].

At present, the most reliable method to propagate GS-cell lines from primary GBM is the selection for cells that grow as neurospheres in the absence of serum and in the presence of EGF and bFGF [67]. These cells then have to be extensively characterized for their capacities for serial self-renewal, differentiation, and* in vivo* tumorigenicity [68]. Although CD133 expression identifies a possible stem-like lineage within GBM, it is not the single universal marker identifying GS-cells [65]. In order to fully recapitulate the cellular heterogeneity of the stem-like compartment of GBM* in vitro*, different isolation approaches and cell culture protocols have to be combined and refined, for example, the modeling of the hypoxic stem-cell niche* in vitro*, which might increase the frequency of isolated GS-cells [20, 22].

## 3. *EGFR*-Amplified Glioblastoma Cells with a Stem-Like Phenotype* In Vitro*

One major shortcoming of the above-mentioned methods is that although GS-cells resemble the genetic and transcriptional phenotype of the original tumor closely [13, 14],* EGFR* amplification as the most frequent molecular alteration is usually not preserved* in vitro* [49].* EGFR* amplification can only be maintained for a limited number of passages* in vitro* (*n* < 5) either using conventional or GS cell culture conditions at normoxia (21% O_2_) or at hypoxia (1% O_2_) [14, 69]. Experimental systems to retain* EGFR* amplification present in the original tumor have thus largely relied on immediate orthotopic implantation of freshly resected tissue from GBM with* EGFR* amplification into nude mice [49, 70, 71] and by subsequent serial passaging* in vivo* of these xenograft tumors [42, 72, 73]. Apparently, the* in vivo* conditions provide a favorable microenvironment for* EGFR*-amplified cells, whereas standard* in vitro* conditions exert a negative selection pressure for those cells [38, 49]. However, after several passages* in vivo* (4-5), the serially transplanted tumors also lose their EGFR overexpression and histologically change from an invasive to a solid, vascularized morphology (“angiogenic switch”). These xenotransplantation approaches, although delivering valuable information, are laborious, time consuming (the time to development of symptoms ranges from 70 to 150 days), difficult to standardize, and limited to analyses* in vivo*. To study* EGFR* amplification* in vitro*, permanent cell lines with endogenous EGFR amplification and with stem-like features, such as self-renewal, clonogenicity, and the potential for* in vivo* tumorigenicity, would be the ideal model system.

In this regard, short-term culturing of GBM-derived primary cells as three-dimensional tumor spheres under stem cell conditions rather than as adherent monolayers has indicated that* EGFR* amplification can be maintained* in vitro* [74–76]. When propagated in the absence of serum, tumor spheroids retained* EGFR* amplification and an associated polysomy of chromosome 7 as determined by FISH analysis. Additionally, heterogeneous EGFRvIII-expression, when present in the original tumor, was preserved* in vitro* as well [77]. Furthermore, tumor-derived spheroids from short-term cultures have been shown to initiate xenograft tumors that phenocopy the EGFR status of the original tumor* in vivo*, even when cultured in the presence of serum on agar-coated cell culture plates in order to avoid attachment [70, 78]. Culturing cells derived from* EGFR*-amplified GBMs as spheroids can conserve* EGFR* aberrations for a limited number of passages, thereby allowing for analyses of EGFR-related processes in a naturally* EGFR*-amplified background* in vitro* and* in vivo*, for example, response to EGFR-targeted therapy with tyrosine kinase inhibitors (TKIs) or monoclonal antibodies (mAbs) [70, 74, 76, 77].

One possible reason for the loss of* EGFR* amplification* in vitro* in addition to the growth pattern* in vitro* (adherent versus spheroid) is the propagation of tumor-derived cells in the presence of exogenous mitogens, especially EGF. EGF has been shown to inhibit the growth of* EGFR*-amplified MDA-468 breast cancer and A431 epidermoid carcinoma cells, which both strongly overexpress EGFR at the protein level [79–81]. In addition, EGF can induce apoptosis by activating the EGFR in A431 cells, which can be abrogated by tyrosine kinase inhibition [82]. Abundance of EGFR signaling due to increased receptor expression and subsequent ligand-induced overstimulation of the EGFR pathway therefore seems to be a major negative selector for* EGFR*-amplified GBM cells* in vitro*.

Following this line of evidence, we recently demonstrated that the modulation of exogenous EGF concentrations and otherwise unaltered neurosphere conditions preserves genetic* EGFR* aberrations in an EGF-dependent manner. Omitting EGF from the cell culture medium when primary GBM cells are taken into culture preserved* EGFR* amplification and EGFRvIII expression with high success rates (approximately 40% of all tumors with* EGFR* amplification taken into culture) [69]. By applying different EGF concentrations (0 to 20 ng/mL), we were able to generate isogenic permanent cell lines from the same tumor with stable* EGFR* amplification and EGFRvIII expression (>15 passages) in the absence of EGF and nonamplified, EGFRvIII-negative cell lines in the presence of 20 ng/mL EGF. This method therefore allows for the conservation of EGFR-dependent intratumoral heterogeneity* in vitro*. The cell lines exhibited a stem-like phenotype; that is, they expressed CD133, showed the capacity for self-renewal, could be differentiated along astrocytic, oligodendrocytic, and neuronal lineages, and recapitulated the heterogeneous EGFR expression of the original tumor when implanted into immunocompromised mice. Importantly, tumorigenicity was enhanced for* EGFR*-amplified cells (median survival 102 versus 117 days, *p* = 0.0018, log-rank test), emphasizing the relevance of EGFR expression for the progression of GBM* in vivo*.

Spontaneous conservation of* EGFR* amplification in permanent cell lines with a stem-like phenotype was however reported occasionally even in the presence of EGF. Mazzoleni et al. described two neurosphere cell lines with stem-like features and heterogeneous* EGFR* amplification maintained under standard neurosphere conditions, termed L0306 and L0627 with low and high* EGFR* amplification, respectively [83]. Importantly, the authors could show that reduction of exogenous EGF led to a reexpression of EGFR protein in cells formerly negative for EGFR. Furthermore, our own data recently indicated that the highly* EGFR*-amplified L0627 could be propagated permanently in the absence of EGF without any changes in proliferation,* EGFR* amplification, or stem-like features, while EGF withdrawal from L0306 led to significantly reduced proliferation [84, 85]. This finding indicates that high level* EGFR* amplification might be a prerequisite for the generation of permanent cell lines from* EGFR*-amplified GBM in the absence of EGF.

## 4. EGFR and a Stem-Like Phenotype in GBM

**Figure 1:**
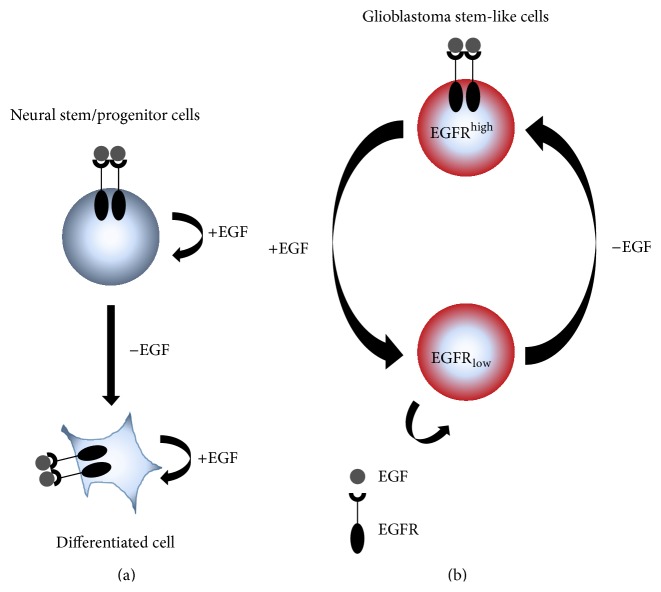
EGF/EGFR-dependent plasticity of neural stem/progenitor cells (NSC) and glioma stem-like cells (GS-cells). (a) In NSCs, EGF promotes self-renewal and proliferation, while withdrawal of EGF leads to terminal differentiation along astrocytic, neuronal, and oligodendrocytic lineages. (b) In GS-cells, EGF modulates the expression of EGFR at the protein level and* EGFR* amplification present in the original tumor. Withdrawing EGF from cell culture can in some cases lead to an upregulation of EGFR expression, while repeated stimulation with exogenous EGF reduces the amount of EGFR in the cells.

The apex cell of the cellular hierarchy of GBM still remains elusive [1, 86, 87]. However, experimental evidence from a genetically engineered mouse brain tumor model that allows for lineage tracking of neural stem/progenitor cells indicates that NSCs have to be considered the prime suspects for the cell of origin in GBM [88]. Furthermore, adult NSCs share key characteristics with GS-cells, such as the capacity for self-renewal and differentiation as well as spherical growth* in vitro* under the same cell culture conditions and a highly migratory phenotype, albeit with different dynamics [1, 86, 89]. Additionally, the reliance on stimulation with specific exogenous growth factors in order to maintain a stem-like phenotype is striking. For NSCs, EGF-induced activation of EGFR increases proliferation, survival, and migration, while inhibiting differentiation, whereas withdrawal of EGF from NSC cultures leads to differentiation and cell death (Figure 1(a)) [90]. For GS-cells, however, we and others could show that proliferation and maintenance of a stem-like phenotype were solely dependent on bFGF and not on EGF, even though GS-cells were still sensitive to stimulation with exogenous EGF and responded with enhanced proliferation and neurosphere size (Figure 1(b)) [69, 74, 91–93]. Furthermore, withdrawal of EGF led to preservation or even a regain of molecular EGFR aberrations and/or EGFR protein overexpression, which is usually lost in the presence of EGF [69, 83, 84]. One possible explanation is that strong overexpression of EGFR renders cells autonomous of exogenous ligand stimulation through ligand-independent mechanisms or spontaneous receptor activation [94–97]. Moreover, GS-cells with conserved* EGFR* amplification and protein overexpression secrete EGF in amounts that are sufficient to stimulate EGFR phosphorylation in an autocrine activation loop [69]. Even relatively small amounts of secreted EGF can activate EGFR signal transduction, since only a single EGF molecule is necessary to activate one EGFR dimer [98].

Within the stem-like compartment of* EGFR*-amplified GBM, EGFR seems to define a distinct cellular hierarchy [83, 99]. By dividing* EGFR*-amplified GS-cells into EGFR^high^ and EGFR_low_ cells by fluorescence activated cell sorting, Mazzoleni et al. could determine an EGFR-dependent cellular hierarchy with distinct molecular and functional phenotypes. The authors described high EGFR expression to confer the highest malignancy to GS-cells. Similarly, we could demonstrate that GS-cells with retained* EGFR* amplification proliferated much faster* in vivo* than GS-cells from the same primary tumor without* EGFR* amplification. These results indicate a higher degree of “stemness” associated with* EGFR* amplification and EGFR overexpression [69, 83].

**Figure 2:**
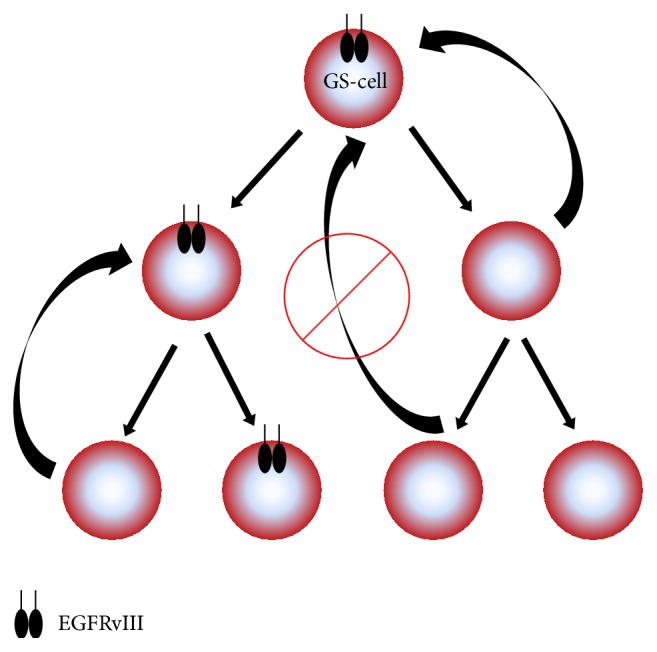
EGFRvIII-dependent hierarchy of glioma stem-like cells (GS-cells). An EGFRvIII-expressing GS-cell at the apex of the cellular hierarchy in GBM can divide into EGFRvIII-positive and negative cells, thereby maintaining the EGFRvIII-positive GS-cell reservoir. Within a limited time frame, EGFRvIII-negative cells can regain EGFRvIII expression, an ability which is lost further downstream in the EGFRvIII-dependent hierarchy [69, 83, 98].

*EGFR* amplification and the* EGFR* gene rearrangement events leading to the loss of exons 2–7 resulting in EGFRvIII expression are considered to be early events in GBM development [99]. In analogy to the unaltered full-length EGFR, EGFRvIII is associated with a cellular hierarchy in EGFRvIII-positive GBM (Figure 2). Interestingly, EGFRvIII-positive cells can give rise to both EGFRvIII-positive and -negative cells. However, reexpression of EGFRvIII can only occur in a cell that has just recently lost EGFRvIII and has not persisted in an EGFRvIII-negative state for increased time duration [83, 99]. It has also been demonstrated that GBM contain a CD133^+^/EGFRvIII^high^ subpopulation of stem-like cells [100]. Additionally, EGFRvIII can keep glioma cells in an undifferentiated, stem-like state whereas differentiation of EGFR^high^/EGFRvIII^+^ GS-cells leads to downregulation of both receptors and a loss of stem-like potential [101, 102]. Vice versa, upregulation of EGFR in a telomerase reverse transcriptase- (TERT-) dependent manner allows differentiated glioma cells to acquire stem-like features [103]. Furthermore, EGFRvIII has been shown to enhance* in vivo* tumorigenicity of GBM cells in cooperation with EGFR, indicating an enhanced stem-like potential in the presence of EGFRvIII [50, 69, 102].

## 5. Targeting Glioma Stem-Like Cells via EGFR/EGFRvIII

*EGFR* amplification and protein overexpression are considered potential therapeutic targets in neurooncology. In particular, the expression of EGFRvIII, which comprises a unique tumor-specific target in approximately 30% of all newly diagnosed GBM, offers many possibilities [38]. However, clinical trials targeting EGFR function have been so far disappointing since the heterogeneous distribution of EGFR throughout the tumor might render cells differentially sensitive towards EGFR inhibition, ultimately leading to therapy failure [51, 69, 70, 83]. Strikingly, EGFRvIII seems to be closely associated with an acquired resistance against targeted therapy with TKIs [104, 105]. Nathanson et al. described an EGFRvIII-positive subpopulation of tumor cells which they isolated from EGFRvIII-expressing GBM patients who developed resistance to TKI-therapy after an initial response [75]. This subpopulation persisted during TKI-treatment and expanded again after drug withdrawal. The authors described this subpopulation of cells to grow as neurospheres* in vitro* and to give rise to highly heterogeneous xenograft tumors, indicating that they possessed stem-like features.

The difficulties with TKI or mAbs targeting EGFR have sparked the development of alternative treatment strategies to exploit EGFR or EGFRvIII as a molecular target in GBM. Current approaches are utilizing EGFR/EGFRvIII as a unique tumor antigen to specifically identify GBM cells rather than targeting the EGFR's biological function and have emphasized the significance of EGFR/EGFRvIII as a target for GBM therapy [106]. Arming the patients' immune system against GBM with* EGFR* amplification and EGFRvIII expression appears to be especially promising. Currently, the most exciting systemic approach to exploit the exclusive expression of EGFRvIII by the tumor is a vaccination strategy with a peptide termed rindopepimut covering the neoepitope of EGFRvIII (i.e., a novel glycine at the exon 1-exon 8 junction) [107]. In a recent Phase II study, this approach could prolong the OS of patients with newly diagnosed EGFRvIII-positive GBM to 21.8 months with a 36-month OS of 26% [108]. Strikingly, nearly all patients had lost expression of EGFRvIII at recurrence [109]. In line with this data, it was found that treatment-naïve GBM patients already exhibit a strong endogenous immune response against EGFR as indicated by a high level of anti-EGFR serum autoantibodies, pointing towards a high immunogenic potential of EGFR [110].

In a different immunotherapeutic approach, T-cells are equipped with chimeric antigen receptors (CARs) recognizing EGFRvIII, which then effectively target EGFRvIII expressing GS-cells* in vitro* and exhibit significant cytotoxicity. CAR-expressing T-cells also infiltrate and kill established EGFRvIII-positive xenograft tumors in mice [111–114]. In a similar approach, Muller et al. recently demonstrated that engineering NK-cells modified with an EGFRvIII-specific CAR to overexpress CXCR4 improves immunotherapy of CXCL12/SDF-1*α*-secreting glioblastoma in mice [115]. These strategies, although not specifically aiming at GS-cells, might also eradicate the stem-like compartment defined by EGFRvIII.

Emlet et al. developed a bispecific CD133/EGFRvIII antibody to specifically target the CD133^+^/EGFRvIII^high^ subpopulation of GBM [100]. In an* in vitro* cellular cytotoxicity assay, this antibody displayed superior toxicity for CD133^+^/EGFRvIII^high^ glioma cells than for CD133^+^ or EGFRvIII^high^ cells alone and also decreased stem-like properties such as self-renewal. Most importantly, the antibody significantly reduced tumorigenicity* in vivo*, most likely via antibody-dependent cellular cytotoxicity similar to cetuximab [70].

As mentioned, high EGFR/EGFRvIII expression designates an aggressive subtype of GS-cells [69, 100]. Therefore, downregulation of these molecules could represent a potential therapeutic strategy for EGFR-positive tumors [99]. Histone deacetylase inhibitors (HDACi) are an exciting class of anticancer agents. They inhibit the removal of acetyl residues from histones by histone deacetylases (HDAC), resulting in an open chromatin structure and increased transcription, including genetic loci that have been silenced during oncogenesis [116]. This leads to reexpression of proapoptotic and differentiation programs, which partially account for the anticancer effects of HDACi [117, 118]. In GS-cells, the HDACi valproic acid (VPA) induced differentiation and as a result decreased the expression of stem cell markers, rendering them more vulnerable to conventional therapy [119]. Importantly, nonneoplastic cells are relatively resistant to cell death induced by HDAC inhibition [120, 121]. Conversely, HDACi have been described to selectively induce transcriptional repression of high copy number genes such as amplified* EGFR* through blockade of RNA-polymerase II-dependent elongation [122]. As a consequence, the expression of EGFR and of EGFRvIII, which is controlled by epigenetic mechanisms in* EGFR*-amplified cells, can be reduced by HDACi such as Trichostatin A (TSA) or suberoylanilide hydroxamic acid (SAHA) in conventional and GS-cells [75, 84, 99]. Furthermore, treatment of cancer cells with either acquired resistance or an inherent tolerance to EGFR TKIs with HDACi could resensitize these cells to the action of the inhibitor [123]. The combined effects of the TKI erlotinib and different HDACi (SAHA, TSA) were independent of cell culture conditions (neurosphere or containing serum), EGFR status (EGFR^−^/EGFRvIII^−^; EGFR^+^/EGFRvIII^−^; EGFR^+^/EGFRvIII^+^) or acquired TKI resistance [69, 84]. This effect of HDACi might affect also the EGFR-dependent stem-like compartment of GBM and sensitize it to conventional, EGFR-targeted therapy.

## 6. Conclusions and Future Prospects

Endogenous amplification of the* EGFR* gene and overexpression of EGFR/EGFRvIII protein have been difficult to study* in vitro* in the past. Optimization of cell culture conditions for stem-like cells from GBM has enabled researchers to maintain* EGFR*-amplified GS-cells with high EGFR expression in combination with or without EGFRvIII expression at the protein level. These cell culture systems facilitated the analyses of the contribution of EGFR/EGFRvIII to a stem-like phenotype, the discovery of an EGFR/EGFRvIII-dependent cellular hierarchy within the stem-like compartment of GBM, and the development of targeted therapy approaches for EGFR/EGFRvIII-positive GS-cells.

The importance of representative model systems of* EGFR*-amplified GBM for research is highlighted by recent reports which described the occurrence of circulating tumor cells (CTCs) in the blood of more than 20% of GBM patients [124–126]. The study by Müller et al. could demonstrate a significant association between an amplification of the* EGFR* gene in the primary tumor and the occurrence of CTCs in the blood. Importantly, these cells displayed preserved* EGFR* amplification. However, the occurrence of CTCs was not significantly associated with OS of the patient cohort. In other cancers than glioma, the ability of tumor cells to disseminate from the primary tumor mass, to remain dormant for many years, and to survive systemic chemotherapy unharmed has been attributed to cancer stem cell properties. Therefore, in* EGFR*-amplified GBM, cells of the EGFR^high^ GS-cell pool might have the ability to extravasate into the blood stream and to potentially give rise to GBM metastases. Support for this notion comes from reports in the literature describing GBM metastases occurring with a relatively high frequency of 10–20% in transplant patients who received organs from GBM patients [127]. As GBM therapy continues to improve, especially for* EGFR*-amplified, EGFRvIII-positive tumors [108], the likeliness of extracranial metastases might increase from sporadic events to a veritable complication for these patients. Therefore, targeting the EGFR^high^ GS-cell compartment could have prospective benefit for GBM patients with* EGFR*-amplified GBM.

## Abbreviations

GBM: Glioblastoma
GS-cell: Glioma stem-like cell
NSC: Neural stem/progenitor cell
EGFR: Epidermal growth factor receptor
HDAC: Histone deacetylase
HDACi: Histone deacetylase inhibitor
TKI: Tyrosine kinase inhibitor
mAb: Monoclonal antibody.
